# Gestational weight gain and pregnancy outcomes: Findings from North Indian pregnancy cohort

**DOI:** 10.1111/mcn.13238

**Published:** 2021-07-06

**Authors:** Ranadip Chowdhury, Nitika ▪, Tarun Shankar Choudhary, Neeta Dhabhai, Pratima Mittal, Rupali Dewan, Jasmine Kaur, Ritu Chaudhary, Anuradha Tamaria, Rajiv Bahl, Sunita Taneja, Nita Bhandari

**Affiliations:** ^1^ Centre for Health Research and Development Society for Applied Studies New Delhi India; ^2^ Knowledge Integration and Translational Platform (KnIT), Centre for Health Research and Development Society for Applied Studies New Delhi India; ^3^ Vardhman Mahavir Medical College and Safdarjung Hospital New Delhi India; ^4^ Department of Maternal, Newborn, Child, Adolescent Health and Aging World Health Organization Geneva Switzerland

**Keywords:** gestational weight gain, length‐for‐age *z*‐score, low birth weight, prematurity, small‐for‐gestational age, stunting

## Abstract

Despite the high prevalence of inadequate gestational weight gain (GWG) and adverse pregnancy outcomes, very few studies have addressed the association between GWG and pregnancy outcomes in South Asia. Our objectives were to estimate the prevalence of GWG during the second and third trimesters within, below and above the Institute of Medicine (IOM) guidelines, and to estimate the effect of the rate and adequacy of GWG on gestational age at the time of delivery, weight, length, length‐for‐age *z*‐score (LAZ), weight‐for‐length *z*‐score (WLZ) and adverse pregnancy outcomes, namely prematurity, small‐for‐gestational age (SGA), low birth weight (LBW), stunting and wasting at birth. We analysed data from the intervention group of the Women and Infants Integrated Interventions for Growth Study (WINGS), which is an ongoing individually randomized factorial design study. Of the 1332 women analysed, 40.2% [95% confidence interval (CI) 37.5 to 42.8] had GWG below the IOM guidelines. For every 100‐g/week increase in GWG, birth weight increased by 61 g, birth length by 0.16 cm, LAZ score by 0.08 SD, WLZ score by 0.14 SD, and gestational age at birth by 0.48 days. Women with GWG below the IOM guidelines had a higher relative risk of adverse pregnancy outcomes (44% for LBW, 27% for SGA, 32% for stunting and 42% for wasting at birth) than women who had GWG within the IOM guidelines, except for prematurity. The association between GWG and LAZ scores at birth was modified by early pregnancy body mass index (BMI). GWG is a strong predictor of newborn anthropometric outcomes and duration of gestation but not prematurity.

**Key messages**

- GWG rate was significantly associated with newborn anthropometric status and gestational age at birth except for prematurity and stunting.
- GWG below the IOM guidelines increased the risk of adverse pregnancy outcomes except for prematurity.
- Association between GWG and LAZ scores at birth was modified by early pregnancy BMI.
- GWG can be used to track nutritional status during pregnancy at the primary care level.

## 1 INTRODUCTION

In South Asia, particularly in India, low birth weight [LBW; prematurity and/or small‐for‐gestational age (SGA)] and stunting at birth are major public health problems (Lee et al., 2017; Prendergast & Humphrey, 2014). In India, the prevalence of LBW, preterm birth, stunting at birth and SGA are 16%, 18%, 15% and 36%, respectively [Lee et al., 2017; International Institute for Population Sciences (IIPS) and Macro International]. These infants are at an increased risk of neonatal and post‐neonatal mortality, suboptimal neurodevelopment and non‐communicable diseases later in life (Kozuki et al., 2013; Lawn et al., 2014).

Demographic surveillance data showed that the average weight gain among normal‐weight pregnant women in India is only around 60% of the recommended weight gain (Coffey, 2015). A systematic review of studies primarily from higher income countries (HICs) showed that 23% of the pregnant women gained less weight than the range recommended by Institute of Medicine (IOM) (Goldstein et al., 2017). However, very few studies addressed the relationship between GWG and pregnancy outcomes in South Asia despite a higher prevalence of inadequate GWG and adverse pregnancy outcomes than those in HICs (Kac et al., 2019).

We are conducting an individually randomized controlled study with a factorial design [Women and Infants Integrated Interventions for Growth Study (WINGS)] that aims to measure the impact of a package of interventions delivered concurrently during pre‐ and peri‐conception, pregnancy and early childhood periods on preterm labour, SGA and stunting at 24 months of child age (Taneja et al., 2020). WINGS provided us with an opportunity to estimate GWG prevalence during the second and third trimesters using the IOM guidelines in a cohort of pregnant women receiving all evidence‐based interventions to improve pregnancy outcomes. We also assessed the association between the rate of GWG and its adequacy as a predictor of different newborn anthropometric outcomes and gestation.

## 2 METHODS

### 2.1 Design

This was a cohort study that utilized data from an ongoing individually randomized controlled study being conducted in urban and peri‐urban low‐to‐mid socio‐economic neighbourhoods of South Delhi, India. The study protocol has been published elsewhere (Taneja et al., 2020).

Briefly, eligible women aged between 18 and 30 years were identified through a door‐to‐door survey. Women who provided consent to participate in the study were enrolled (first randomization; to pre‐ and peri‐conception intervention or routine care group) and followed up until their pregnancies were confirmed or 18 months after enrolment was completed. Once pregnancy was confirmed by ultrasonography, consent was obtained (second randomization; to enhance antenatal, postnatal, and early childhood care or routine antenatal, postnatal, and early childhood care groups) from the women for participation in the study. For the current analysis, we included pregnant women from the intervention group with the following characteristics: singleton pregnancy delivered until 20 March 2020, gestational age ≤ 20 weeks at the time of second randomization, and at least one weight measurement in the third trimester (≥28 weeks of gestation).

Women in the pre‐ and peri‐conception intervention group received interventions in health, nutrition, psychosocial care and the WASH domain so that they were infection‐free, nutritionally replete and in a positive mental health state when they became pregnant. Women in the control group sought routine care from usual government sources (free of cost) and private providers.

All pregnant women received 180 ml of milk (70 kcal, 6‐g protein) 6 days a week throughout pregnancy from community workers who visited the participants and observed the intake. Women with body mass index (BMI) < 25 kg/m^2^ were given locally prepared snacks throughout pregnancy to cover the recommended additional requirements (280 kcal, 8‐g protein in the second trimester; and 470 kcal, 27‐g protein in the third trimester). Monthly weight measurements were taken at home or at the outreach clinic, and women with GWG below the IOM guidelines were given one hot‐cooked meal (500 kcal, 20‐g protein) daily until delivery [Institute of Medicine (IOM), 2009]. Additionally, a pre‐mix with additional calories and protein was given to pregnant women with BMI < 18.5 kg/m^2^. All pregnant women received approximately one recommended daily allowance of micronutrient supplementation daily throughout pregnancy. For antenatal care, the collaborating hospital followed the World Health Organization (WHO) recommendations on antenatal care [World Health Oganization (WHO), 2016]. Women with hypertension, gestational diabetes, severe anaemia and previous bad obstetric history were encouraged to visit the hospital for antenatal care throughout pregnancy. Women were screened for depressive symptoms using the Patient Health Questionnaire (PHQ‐9) (Kochhar et al., 2007) and were sent to a counsellor if the PHQ‐9 score was ≥10. If the score was ≥15 or if the woman had suicidal ideation, she was referred to the hospital or to a psychiatrist. Home‐based counselling by a worker was carried out using a module developed from an adaptation of the WHO Thinking Healthy Module to promote positive thinking and problem‐solving skills (WHO, 2015). Women were also counselled against the use of tobacco (smoke and smokeless form) and advised on ways to reduce exposure to second‐hand smoke.

The WASH interventions during this period included counselling on personal and hand hygiene as well as the use of safe drinking water. Water filters were installed in the homes if the family had not already used one, and food‐grade plastic bottles were given to store water. A handwashing station, if not already available, was also set up. Soap and disinfectant supplies were replenished.

Women in the control group sought routine care from usual government sources (free‐of‐cost) and private providers during pregnancy.

### 2.2 Variable definitions

The exposure variable (GWG) during the second and third trimesters (in kg) was calculated by subtracting the weight at the time of the second randomization (≤20 weeks) from the last recorded weight before delivery. GWG rate (in g/week) was calculated by dividing the total second and third trimester weight gain by the number of weeks between the two weight measurements. GWG rate adequacy (a categorical variable) was defined using the IOM guidelines (IOM, 2009). The cut‐offs used for GWG below the IOM guidelines were as follows: <0.44 kg/week for underweight women (BMI < 18.5 kg/m^2^), <0.35 kg/week for normal‐weight women (BMI 18.5 to 24.99 kg/m^2^), <0.23 kg/week for overweight women (BMI 25 to 29.99 kg/m^2^), and <0.17 kg/week for obese women (BMI > 30 kg/m^2^). The criteria used to define GWG within the IOM guidelines were as follows: 0.44 to 0.58 kg/week for underweight, 0.35 to 0.50 kg/week for normal weight, 0.23 to 0.33 kg/week for overweight, and 0.17 to 0.27 kg/week for obese women. GWG above the IOM guidelines was defined as follows: >0.58 kg/week for underweight, >0.50 kg/week for normal weight, >0.33 kg/week for overweight, and >0.27 kg/week for obese women.

Weight and length were measured by a well‐trained study team within 7 days of birth. Length‐for‐age *z*‐score (LAZ), weight‐for‐length *z*‐score (WLZ) and weight‐for‐age *z*‐score (WAZ) were calculated using the WHO (2006) growth standards (WHO, 2006). Stunting and wasting were defined as LAZ and WLZ scores < −2 SD, respectively. LBW was defined as birth weight less than 2500 g, and the gestational age at birth was calculated by subtracting the date of birth from the date of dating ultrasound and adding it to the estimated gestational age at the time of dating ultrasound using INTERGROWTH‐21 standards (Ioannou et al., 2013; Papageorghiou et al., 2016). Prematurity was defined as birth occurring before 37 weeks of gestation. Spontaneous prematurity was defined as birth occurring before 37 weeks of gestation with preterm premature rupture of membranes or spontaneous onset of labour. Birth weight percentiles were calculated using the INTERGROWTH‐21 standards based on weight measured at any time up to 7 days of birth and gestational age at birth. SGA was defined as birth weight <10th percentile using the INTERGROWTH‐21 standard (Villar et al., 2014).

### 2.3 Statistical analyses and sample size

The available sample size had at least 80% power to detect a relative risk (RR) of 1.5% between GWG below the IOM guidelines and adverse pregnancy outcomes, assuming an adverse pregnancy outcome prevalence between 15% and 35% among women with GWG within IOM guidelines with a 5% significance level (Kac et al., 2019). The available sample was also sufficient to detect a linear regression slope (or beta coefficients) of at least 0.076 between the GWG rate and continuous pregnancy outcomes, assuming 80% power and 5% significance level.

**Figure 1:**
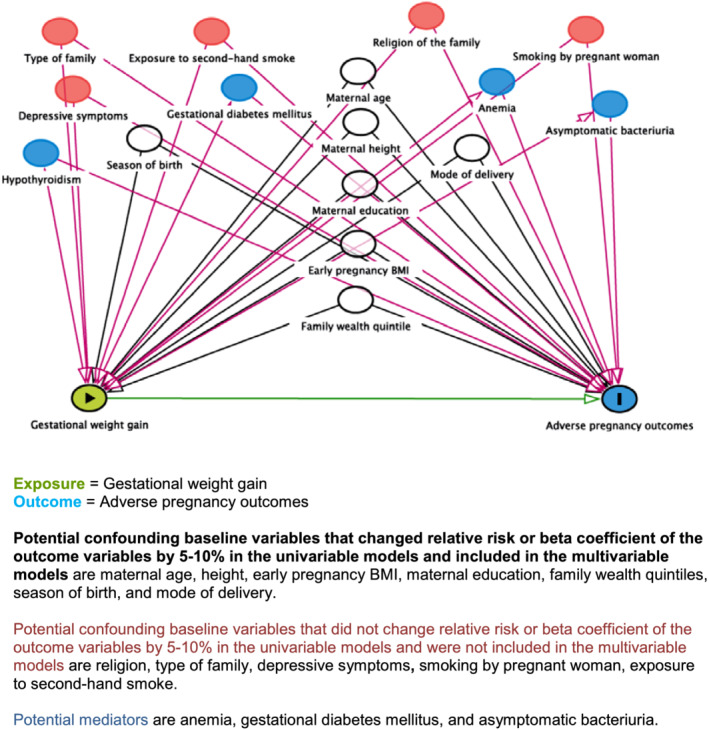
Directed acyclic graph (DAG)

Sociodemographic and clinical characteristics were reported as mean (SD), median [interquartile range (IQR)] or proportion, as appropriate. We used generalized linear models (GLMs) of the Gaussian family with an identity‐link function to calculate the difference in means and 95% confidence intervals (CIs) for continuous outcomes. We used the GLM of the binomial or Poisson family with a log‐link function to calculate the RR and 95% CI for binary outcomes. We used the Poisson family, where the models did not converge using the binomial family (Zou, 2004). We developed a directed acyclic graph (DAG, http://www.dagitty.net/) model to identify potential confounders and mediators based on the literature (Figure 1). The candidate variables were maternal education (none, primary, secondary and higher than secondary), religion (Hindu and others), type of family (extended or joint, and nuclear), family wealth quintiles, maternal height, maternal age, early pregnancy (gestational age ≤ 20 weeks), BMI, anaemia (Hb < 11 g/dl), hypothyroidism (TSH > 5.5 IU/ml), gestational diabetes mellitus (2‐h blood sugar > 140 mg/dl after oral glucose tolerance test using 75‐g glucose), asymptomatic bacteriuria (urine culture with >10^5^ colony forming units), depressive symptoms using PHQ‐9, smoking, exposure to second‐hand smoke, season of birth (monsoon: June, July, August and September; summer: March, April and May; and winter/pre‐winter: October, November, December, January and February), and type of delivery (normal, assisted vaginal or caesarean section). The family wealth index was calculated for each participant by performing a principal component analysis based on all 33 assets owned by the household as done in national surveys [International Institute for Population Sciences (IIPS) and Macro International]. The total scores were used to divide the population into five equal wealth quintiles: the poorest, very poor, poor, less poor and least poor. We used a purposeful selection of covariates to identify variables for the multivariable models (Bhavadharini et al., 2017; Gondwe et al., 2018). For this, we included variables that changed the RR or beta coefficient of the outcome variables by 5%–10% in the univariable models. We presented both unadjusted and adjusted effect sizes, including variables that were identified in the process.

The variables that changed RM or beta coefficient of the outcome variables by 5%–10% in the univariable models and were included in the multivariable models were maternal age, height, early pregnancy BMI, maternal education, family wealth quintiles, season of birth and mode of delivery. The variables that did not change RR or beta coefficient of the outcome variables by 5%–10% in the univariable models were not included in the multivariable models; these included religion, type of family, depressive symptoms, smoking by pregnant woman and exposure to second‐hand smoke. The mediator variables were anaemia, gestational diabetes mellitus and asymptomatic bacteriuria.

We assessed whether early pregnancy BMI (underweight, normal weight and overweight or obese) modified the association between second and third trimester GWG rates with outcomes by adding an interaction term. The outcome with significant interaction (*p* < 0.05) was examined with stratified analyses, and separate regression lines were estimated to examine the interaction. All **s**tatistical analyses were performed using STATA version 16 (StataCorp, College Station, TX, USA).

### 2.4 Ethical considerations

The Ethics Review Committees of the Society for Applied Studies, Vardhman Mahavir Medical College and Safdarjung Hospital, and the World Health Organization, Geneva, have approved the study.

## 3 RESULTS

In the intervention group of WINGS, 1453 pregnant women delivered a live‐born baby before the initiation of the analysis. Using the inclusion criteria of singleton pregnancy, gestational age ≤ 20 weeks at the time of second randomization, and at least one weight measurement in the third trimester (≥28 weeks of gestation), 1332 women were included in the analysis.

The sociodemographic characteristics of the pregnant women included (*n* = 1332) in the analysis were similar to those (*n* = 121) not included, except for height (152.6 vs. 150.4 cm; *p* < 0.001) and annual household income (USD 3093 vs. USD 2651; *p* = 0.03), which were higher in the analytical cohort.

Table 1 shows the sociodemographic and clinical characteristics of the pregnant women included in the analysis. At the time of enrolment, 16.1% of the pregnant women were underweight (BMI < 18.5 kg/m^2^), 18.8% were overweight (BMI ≥ 25 to 29.9 kg/m^2^), and 4.1% were obese (BMI ≥ 30 kg/m^2^). Approximately, 30% of pregnant women had short stature (height < 150 cm), and 50% had completed 12 or more years of education. The mean (SD) gestational age at the time of reporting of pregnancy and the last weight measured before delivery was 11.2 (1.9) and 37.7 (1.7) weeks, respectively. The median (IQR) difference between the time of delivery and the last weight measured was 5 (3–7) days.

**Table 1:** Sociodemographic and clinical characteristics of pregnant women

	*n* = 1332
Characteristics of pregnant women	
Age (years), mean ± SD	23.7 ± 3
Height (cm), mean ± SD	152.6 ± 5.7
Height < 150 cm	435 (32.7)
Years of schooling	
None (0)	58 (4.4)
Primary (1–5)	118 (8.9)
Secondary (6–12)	513 (38.5)
Higher than secondary (>12)	643 (48.3)
Occupation	
Housewife	1264 (94.9)
Early pregnancy BMI	
Underweight (<18.5 kg/m^2^)	215 (16.1)
Normal weight (18.5 to 24.9 kg/m^2^)	812 (61.0)
Overweight (≥25 to 29.9 kg/m^2^)	251 (18.8)
Obese (≥30 kg/m^2^)	54 (4.1)
Morbidities during pregnancy	
Anaemia	298 (22.4)
Hypothyroidism	117 (8.8)
Gestational diabetes	190 (14.3)
Asymptomatic bacteriuria	264 (19.8)
Moderate to severe depression or suicidal thoughts	46 (3.4)
Smoking during pregnancy	7 (0.5)
Exposure to second‐hand smoke	270 (20.3)
**Family characteristics**	
Religion of head of the household	
Hindu	1102 (82.7)
Annual household income in USD, mean ± SD	3092.1 ± 1678.4
Wealth quintiles	
Poorest	190 (14.3)
Very poor	279 (20.9)
Poor	301 (22.6)
Less poor	260 (19.5)
Least poor	302 (22.7)
Family structure	
Extended or joint^a^	920 (69.1)

*Note*: All values are numbers (percentages) unless stated otherwise.

Abbreviation: BMI, body mass index.

^a^ Extended family: Family unit living with parents, their children, and other dependent blood relatives; joint family: family unit living with grandparents, parents, and their children living together in a household.

**Figure 2:**
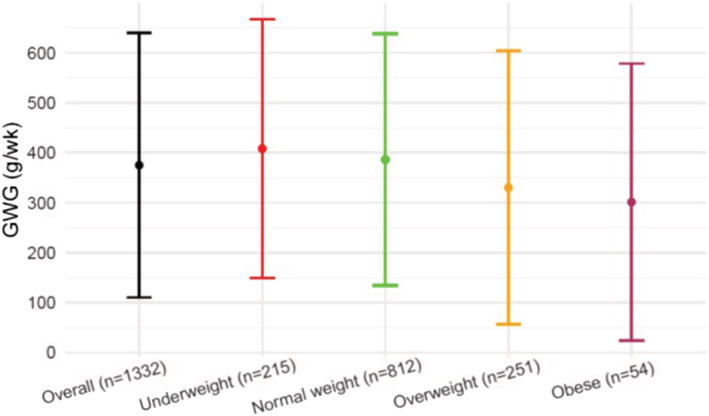
Gestational weight gain rate (g/week) during the second and third trimesters according to early pregnancy body mass index (BMI). GWG, gestational weight gain

Figure 2 shows the rate of GWG (g/week) during the second and third trimesters, overall, and according to BMI categories. The overall mean (SD) GWG rate was 375.9 (134.8) g/week. The mean (SD) GWG rate was 408 (132.1), 386.4 (128.5), 330.1 (139.7) and 301.2 (141.3) g/week in underweight, normal‐weight, overweight and obese women, respectively.

**Figure 3:**
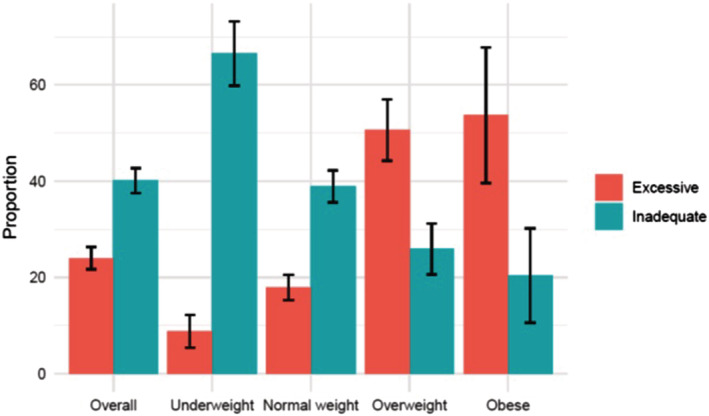
GWG below and above the IOM guidelines during the second and third trimesters using the IOM guidelines according to early pregnancy body mass index (BMI). GWG, gestational weight gain; IOM, Institute of Medicine

Figure 3 shows the proportion of pregnant women with GWG below and above the IOM guidelines. The overall prevalence of GWG below the IOM guidelines was 40.2% (95% CI, 37.5 to 42.8). The proportion of women with GWG below the IOM guidelines was highest (66.5%) among underweight women, followed by 38.9%, 25.9% and 20.4% among normal‐weight, overweight and obese women, respectively. The proportion of women with GWG within the IOM guidelines was similar among underweight (24.6%), overweight (23.5%) and obese (25.9%) women, and higher in normal‐weight (43.2%) women. The proportion of women with GWG above the IOM guidelines was higher among overweight and obese women than among underweight or normal‐weight women (50.6% and 53.7% vs. 8.8% and 17.9%, respectively).

Table 2 shows the association between the second and third trimester GWG rates and pregnancy outcomes. Multivariable analyses showed that for every 100 g/week increase in GWG rate, birth weight increased by 61 g, birth length by 0.16 cm, LAZ score by 0.08 SD, WLZ score by 0.14 SD, and gestational age at birth by 0.48 days. There was a 17% reduction in the risk of LBW and wasting, and 13% risk reduction for SGA for every 100 g/week increase in GWG. However, there was no significant association between prematurity (overall and spontaneous) and GWG rate. There was a reduction in the risk of stunting by 8% for every 100 g/week increase in GWG, but this effect did not reach statistical significance.

**Table 2:** Association between rate of gestational weight gain (100 g/week) during the second and third trimesters with pregnancy outcomes

	Unadjusted *β* coefficient (95% CI)	Adjusted *β* coefficient^b^ (95% CI)
Birth weight (g)	49.49 (33.73, 65.25)	61.00 (45.00, 76.96)
Birth length (cm)	0.14 (0.07, 0.22)	0.16 (0.08, 0.24)
Length‐for‐age *z*‐score at birth	0.07 (0.03, 0.11)	0.08 (0.04, 0.12)
Weight‐for‐length *z*‐score at birth^a^	0.10 (0.06, 0.14)	0.14 (0.10, 0.18)
Weight‐for‐age *z*‐scores at birth	0.12 (0.08, 0.15)	0.14 (0.10, 0.18)
Gestational age at birth (days)	0.54 (0.17, 0.91)	0.48 (0.08, 0.88)

	Unadjusted RR (95% CI)	Adjusted RR^a^ (95% CI)
Low birth weight	0.86 (0.80, 0.91)	0.83 (0.76, 0.90)
Small‐for‐gestational age	0.91 (0.86, 0.96)	0.86 (0.80, 0.93)
Prematurity (overall)	0.94 (0.84, 1.06)	0.98 (0.86, 1.12)
Prematurity (spontaneous)	0.94 (0.81, 1.10)	0.94 (0.78, 1.12)
Stunting at birth	0.94 (0.86, 1.03)	0.92 (0.83, 1.03)
Wasting at birth^a^	0.89 (0.80, 0.99)	0.84 (0.73,0.95)

Abbreviations: CI, confidence interval; RR, relative risk.

^a^ The sample size for weight‐for‐length *z*‐score and wasting outcome was 1242. Weight‐for‐length *z*‐scores could not be calculated using WHO standards as length was <45 cm.

^b^ Adjusted for maternal age, maternal height, early pregnancy body mass index (BMI), education, wealth quintiles, and season of birth.

**Figure 4:**
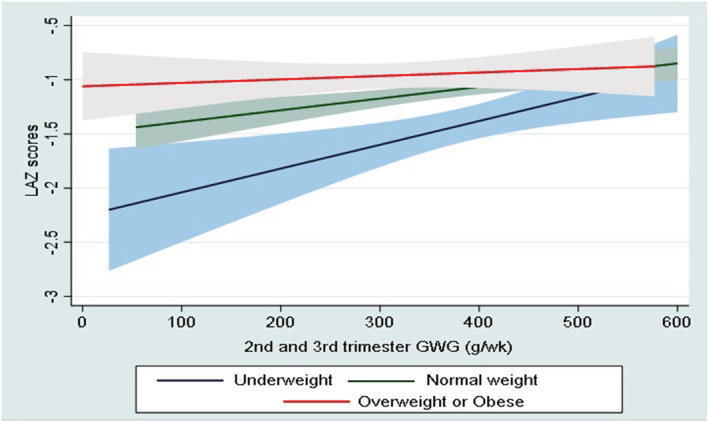
Relationship between second and third trimester gestational weight gain and length‐for‐age *z*‐score (LAZ) at birth stratified by early pregnancy body mass index (BMI). *p* for interaction = 0.028. The blue line indicates underweight (BMI < 18.5 kg/m^2^), the green line shows normal weight (BMI 18.5 to 24.9 kg/m^2^) and the red line indicates overweight or obese (BMI ≥ 25 kg/m^2^). The bands represent 95% confidence intervals (CIs) from the linear regression model. Beta coefficients and 95% CI for underweight, normal weight and overweight or obese pregnant women were 0.17 SD; 95% CI, 0.06 to 0.27, 0.07, SD; 95% CI, 0.02 to 0.12 and 0.04 SD; 95% CI −0.04 to 0.12, respectively. GWG, gestational weight gain

Tables 3 and 4 show the association between GWG adequacy during the second and third trimesters with pregnancy outcomes. There was a significant decrease in all the continuous pregnancy outcomes (birth weight by −127 g, LAZ score −0.18 SD, WLZ score −0.27 SD and gestational age at birth by −1.18 days) among women with GWG below the IOM guidelines compared with women with GWG within the IOM guidelines. Similarly, there was an increased RR for all adverse pregnancy outcomes in pregnant women with GWG below the IOM guidelines compared with those with GWG within the IOM guidelines (44% for LBW, 27% for SGA, 32% for stunting and 42% for wasting at birth) except for prematurity (both overall and spontaneous). However, there was no significant association between GWG above the IOM guidelines and pregnancy outcomes. The association between GWG and LAZ score was modified by early pregnancy BMI (*p* value for interaction = 0.028). The relationship between newborn LAZ and GWG rate was steeper among underweight women than among overweight or obese women. The mean LAZ score of babies born to underweight women was similar to that of babies born to women with normal weight or overweight or obese women if the second and third trimester GWG rates were >500 g/week (Figure 4). The mean LAZ scores of babies born to overweight or obese women were approximately −1 SD throughout the second and third trimester GWG (Figure 4).

**Table 3:** Associations between gestational weight gain adequacy during the second and third trimesters with birth outcomes

	GWG below IOM guidelines (*n* = 535)	GWG within IOM guidelines (*n* = 477)	GWG above IOM guidelines (*n* = 320)	*p* value*	GWG below IOM guidelines^a^		GWG above IOM guidelines^a^	
					Unadjusted	Adjusted^b^	Unadjusted	Adjusted^b^
Birth weight (g)	2618.57 ± 383.05	2764.10 ± 376.75	2856.76 ± 418.01	<0.001	−145.53 (−193.61, −97.45)	−126.83 (−173.88, −79.79)	92.66 (37.49, 147.83)	50.41 (−5.10, 105.91)
Birth length (cm)	47.52 ± 1.96	47.96 ± 1.78	48.20 ± 1.96	<0.001	−0.44 (−0.67, −0.20)	−0.37 (−0.60, −0.14)	0.24 (−0.03, 0.50)	0.09 (−0.18, 0.36)
LAZ score at birth	−1.24 ± 1.04	−1.02 ± 0.92	−0.89 ± 1.00	<0.001	−0.22 (−0.34, −0.10)	−0.18 (−0.30, −0.06)	0.13 (−0.01, 0.27)	0.05 (−0.09, 0.19)
WLZ score at birth^c^	−1.14 ± 0.90	−0.84 ± 0.93	−0.64 ± 0.97	<0.001	−0.30 (−0.42, −0.18)	−0.27 (−0.39, −0.15)	0.20 (0.07, 0.34)	0.13 (−0.01, 0.26)
WFA *z*‐score at birth	−1.55 ± 0.94	−1.20 ± 0.87	−0.99 ± 0.95	<0.001	−0.34 (−0.46, −0.23)	−0.30 (−0.40, −0.19)	0.21 (0.08, 0.34)	0.10 (−0.02, 0.24)
Gestational age at birth in days	269.16 ± 9.66	270.40 ± 9.00	269.91 ± 9.52	0.01	−1.24 (−2.39, −0.08)	−1.18 (−2.35, −0.01)	−0.48 (−1.81, 0.85)	−0.43 (−1.80, 0.95)

*Note*: Data are mean ± SD or *β* coefficient (95% CI).

Abbreviations: GWG, gestational weight gain; IOM, Institute of Medicine; LAZ, length‐for‐age *z*‐score; WFA, weight‐for‐age *z*‐scores; WLZ, weight‐for‐length *z*‐score.

^a^ Reference category: adequate gestational weight gain.

^b^ Adjusted for maternal age, height, early pregnancy BMI, education, wealth quintiles, season of birth and type of delivery.

^c^ Sample sizes for weight‐for‐length *z*‐score outcomes were 457, 484 and 301 in adequate, inadequate and excessive weight groups, respectively.

^*^ *p* value estimated using ANOVA.

**Table 4:** Association between gestational weight gain adequacy during second and third trimester and adverse pregnancy outcomes

	GWG below IOM guidelines (*n* = 535)	GWG within IOM guidelines (*n* = 477)	GWG above IOM guidelines (*n* = 320)	*p* value^*^	GWG below IOM guidelines^a^		GWG above IOM guidelines^a^	
					Unadjusted	Adjusted^b^	Unadjusted	Adjusted^b^
LBW	196 (36.6)	114 (23.9)	66 (20.6)	<0.001	1.53 (1.26, 1.86)	1.44 (1.14, 1.82)	0.86 (0.66, 1.13)	0.95 (0.70, 1.30)
SGA	255 (47.7)	167 (35.0)	74 (23.1)	<0.001	1.36 (1.17, 1.58)	1.27 (1.04, 1.55)	0.66 (0.52, 0.83)	0.76 (0.58, 1.01)
Prematurity (overall)	60 (11.2)	54 (11.3)	32(10.0)	0.82	0.99 (0.70, 1.40)	0.92 (0.63, 1.33)	0.88 (0.58, 1.33)	0.96 (0.61, 1.50)
Prematurity (spontaneous)	38 (6.7)	33 (6.9)	14 (4.4)	0.29	0.97 (0.62, 1.53)	0.89 (0.55, 1.43)	0.63 (0.34, 1.16)	0.74 (0.39, 1.40)
Stunting	104 (19.4)	69 (14.5)	46 (14.4)	0.04	1.34 (1.02, 1.77)	1.32 (1.01, 1.71)	0.99 (0.70, 1.40)	1.02 (0.70, 1.49)
Wasting^c^	78 (16.1)	51 (11.2)	27 (9)	0.007	1.44 (1.04, 2.00)	1.42 (1.01, 2.01)	0.80 (0.51, 1.25)	0.82 (0.51, 1.31)

*Note*: Data are *n* (%) or relative risk, RR (95% CI).

Abbreviations: GWG, gestational weight gain; LBW, low birth weight; SGA, small‐for‐gestational age.

^a^ Reference category: adequate gestational weight gain.

^b^ Adjusted for maternal age, height, early pregnancy BMI, education, wealth quintiles and season of birth.

^c^ Sample sizes for wasting outcomes were 457, 484 and 301 in adequate, inadequate and excessive weight groups, respectively.

^*^ *p* value estimated using the chi‐square test.

## 4 DISCUSSION

In this pregnancy cohort, the mean (SD) GWG in the second and third trimesters was 9.9 (3.7) kg, and 40.2% of pregnant women had GWG below the IOM guidelines. The GWG rate was significantly associated with anthropometry and gestational age at birth but was not associated with prematurity or stunting at birth. GWG below the IOM guidelines increased the risk of adverse pregnancy outcomes compared with GWG within IOM guidelines except for prematurity.

We found that the mean GWG rate was higher and prevalence of GWG below the IOM guidelines was lower during the second and third trimesters than those in other low‐income countries (Abdulmalik et al., 2019; Asefa et al., 2020; Bhavadharini et al., 2017; Chen, Zhou, et al., 2020; Gondwe et al., 2018; Hasan et al., 2018; Kac et al., 2019; Tran et al., 2019). In Bangladesh, the mean GWG from enrolment to 36 weeks was 6.5 kg, and 74% of the pregnant women had GWG below the IOM guidelines (Kac et al., 2019). In rural Malawi, 71.8% of pregnant women had GWG below the IOM guidelines (Hasan et al., 2018). A potential explanation for the lower prevalence in our study could be that the women received evidence‐based interventions known to improve pregnancy outcomes and underweight women received additional food until delivery. A systematic review of HICs showed that 23% of pregnant women had GWG below the IOM guidelines, which is almost half of our estimate (Goldstein et al., 2017). However, the prevalence of underweight pregnant women in our study was 16.1% compared with 7% in the systemic review.

Our findings of GWG below the IOM guidelines as an important risk factor for adverse pregnancy outcomes align with previous studies from low‐ and middle‐income countries (Abdulmalik et al., 2019; Bhavadharini et al., 2017; Gondwe et al., 2018; Hasan et al., 2019; Hung et al., 2015; Kac et al., 2019). Similar to our findings, in Bangladesh, women with GWG within the IOM guidelines had a lower risk of adverse pregnancy outcomes (Kac et al., 2019). Women with low weekly GWG had a higher risk of delivering LBW infants than those with normal GWG in rural Malawi (Gondwe et al., 2018). In Vietnam, women with a total GWG of less than 10 kg had 90% increased odds of having SGA babies compared with those with a GWG of 10–15 kg (Young et al., 2017). Very few studies have shown GWG below the IOM guidelines and increased risk of lower LAZ scores and stunting (Gondwe et al., 2018; Kac et al., 2019; Tran et al., 2019).

We found an association of GWG rate with gestational age at delivery but not with prematurity (overall or spontaneous). Other studies have shown mixed results for this association (Chen, Chen, & Hsu, 2020; Dahly et al., 2018; Enomoto et al., 2016; Huang et al., 2018; Shin & Song, 2015). An explanation for this could be that studies showing an association between the rate of GWG and the duration of gestation or prematurity did not adjust for the gestational age at the time that the last weight measurement was taken (Mitchell et al., 2016; Sharma et al., 2015). Women with premature babies have less time to gain weight during pregnancy than women with term babies; thus, the association between total GWG and prematurity could be due to reverse causation. Therefore, weekly GWG seems to be more appropriate for assessing this association.

The association between GWG and LAZ scores at birth was modified by early pregnancy BMI. These were higher in underweight pregnant women (0.17 SD; 95% CI, 0.06 to 0.27) than in overweight or obese women (0.04 SD; 95% CI −0.04 to 0.12). The relationship between the GWG and LAZ scores was stronger among underweight women. Therefore, this subgroup may benefit more from interventions aimed at improving GWG. This is also consistent with the findings of a recent meta‐analysis that showed a stronger association between inadequate GWG and SGA in underweight pregnant women (Goldstein et al., 2017).

In 2009, the IOM published revised GWG guidelines that were based on American women primarily on the basis of primigravid mothers of high socio‐economic status with no physical activity (Rasmussen et al., 2009). Thus, generalizability to other regions, especially to South Asian countries, is unclear as maternal anthropometry varies across different populations (Kelly et al., 1996). Moreover, there are no national guidelines for monitoring GWG in India. The IOM guidelines may not be feasible for use in India as it will be difficult to compare, translate, or generalize the findings.

Our study has a few limitations. First, BMI could not be assessed earlier than the 14th week of gestation for approximately 10% of pregnant women; this may have resulted in incorrect classification of early pregnancy BMI status. Second, women included in the analysis were taller and had higher annual household incomes than those excluded from this analysis, which may have attenuated the effect of GWG on pregnancy outcomes. Third, we did not consider multiple testing analytical strategy as outcomes of interest were closely related (newborn anthropometry and gestation), and all analyses and estimates are reported with equal emphasis.

The strengths of the analysis include a robust assessment of gestational age by ultrasonography, collection of infant and women anthropometric data by a well‐trained team, use of different matrices for GWG, and inclusion of all possible newborn anthropometric outcomes and gestational parameters. Finally, the women were from an intervention group of ongoing randomized controlled trial and received all interventions known to reduce adverse pregnancy outcomes. It is also noteworthy that the unadjusted and adjusted models had similar results, indicating that confounding was minimal. However, there is a possibility that unmeasured confounders may have led to some bias.

## 5 CONCLUSIONS

These results suggest that GWG below the IOM guidelines is a strong predictor of newborn anthropometric outcomes and duration of gestation but not prematurity, both overall and spontaneous, in a North Indian pregnancy cohort. The association between GWG and LAZ scores at birth was modified by early pregnancy BMI.

There was a difference between the rates of early pregnancy underweight status (16.1%) and GWG below the IOM guidelines GWG (40.3%), suggesting that factors other than early pregnancy nutritional status also influence GWG.

## CONFLICTS OF INTEREST

None of the authors have any conflict of interest to declare.

## FUNDING INFORMATION

The study is funded by the Biotechnology Industry Research Assistance Council (BIRAC), Department of Biotechnology, Government of India under the Grand Challenges India—All Children Thriving Initiative (GCI‐ACT Ref BIRAC/GCI/0085/03/14‐ACT) and the Bill and Melinda Gates Foundation, USA (Grant ID #OPP1191052). The funding agencies did not play any role in study design and are neither involved in nor have any influence over the collection, analyses or interpretation of data.

## CONTRIBUTIONS

RC, N, TSC and ST were involved in conceptualizing research questions, preparation of data file, statistical analysis, data interpretation, manuscript writing, editing and finalization. ND, PM, RD, JK, RC, AT, RB and NB were involved in revising the manuscript critically for important intellectual content. All authors have read and approved the final manuscript.

## CLINICAL TRIAL REGISTRATION

Clinical Trial Registry—India, #CTRI/2017/06/008908 (http://ctri.nic.in/Clinicaltrials/pmaindet2.php?trialid=19339%26EncHid=%26userName=societyforappliedstudies).

## Data Availability

The Society for Applied Studies, India is a collaborator in the Healthy Birth, Growth, and Development Knowledge Integration (HBGDKi) initiative launched by the Bill & Melinda Gates Foundation. The data generated from the study will be shared with the HBGDKi repository (https://github.com/HBGDki). However, individual requests can be considered on a case‐by‐case basis. The request for data along with the detailed proposal describing the intended scientific question(s) to be addressed, should be submitted to Dr Sunita Taneja (sunita.taneja@sas.org.in).
